# Mr. Dendy on the Treatment of the Dying

**Published:** 1848-01-01

**Authors:** W. C. Dendy

**Affiliations:** PRESIDENT OF THE MEDICAL SOCIETY OF LONDON, SURGEON TO THE ROYAL INFIRMARY FOR CHILDREN, ETC.


### ON THE PHYSIOLOGY OF DEATH AND THE TREATMENT OF THE DYING.

BY W. C. DENDY, ESQ.

PRESIDENT OF THE MEDICAL SOCIETY OF LONDON, SURGEON TO THE ROYAL INFIRMARY FOR CHILDREN, ETC.

THE sacred psychologist defines death to be the emancipation of the soul from the body. The philosopher, who maintains that there is no life except as the consequence of organization, defines death to be the mere cessation of vitality, and the ultimate chemical decomposition of the organic atoms of the body, and their resolution into their primitive elements—a mere negation of vitality.

The physiologist cannot thus rest satisfied. It is his duty to bring to his aid the beautiful evidence of the laws of the animal economy, by which the Creator works his fiat. He will, therefore, analyse these laws, and make his deductions, and thus

"Look through Nature up to Nature's God."

He must leave *chiefly* to the divine psychologist the question of a spiritual super-addition to structure; nor, however deeply interesting the subject to all, need he *reason* on the identity of soul and mind, since no human intellect, unless specially inspired, is competent to form a clear decision on the subject. The sacred terms, "living soul," or "quickening spirit," must be left out of his category, though he be convinced of the sublime truth, that it is intimately associated with the body, and responsible for its deeds. To attempt to *prove* or disprove this identity would be the height of presumption. We cannot, in the face of revelation, doubt it, for such scepticism would reduce the immortal spirit to a nonenity: its office would be merely to regard the thoughts and deeds of another essence, over which it had no control—for the crimes of which, however, eternal penalties were denounced. If separate or distinct, one or other must be passive.

The inspired writings have taught us the truth; and, moreover, that when the body dies, the spirit is endowed with separate existence. But physiology can, of course, offer no *demonstration* of the flight of the soul; her studies are confined to its tenement of clay; and when she has traced vitality to its visible close, she must leave the cadaverous mass to the process of putrefaction and the worm.

It may, at first, seem that the phrase *physiology* of death is a paradox; that since mortality is so constantly the result of disease, *pathology* should be the word. But death is as natural to man as life, and has its appointed term, even if uninfluenced by disease. In contrast to inorganic matter, man lives, and cannot dissolve or cease, except he die. The Deity as surely doomed his creatures to the one, when he said "thou shalt surely die," as to the other, when he imparted the breath of life, and man became a living soul. Thus we have the term *natural death*, the visitation of God.

We may believe that our first parents would have *slept* and died, had they not erred; what would otherwise have been their change from life we cannot conceive; but we may believe that the effect of sin, as it entailed a life of sorrow and painful parturition, might condemn the human race to an agonizing death.

The unimpeded course of disease, according to natural or pathological law, would ever end in death. Although we may be many years in dying, it is certain that the body is tending towards decay even from the hour of our birth; that influences are incessantly at work to modify and impair the human frame, and render it so far inapt for that gradual and quiet dissolution—a senile euthanasia, the "mors sine morbo" of Richter. It is probable that a *purely healthy* system has never long existed; yet we have records—rare, it is true—of such extreme longevity, so far beyond the appointed hour of the majority of mankind, and that marked by so high a degree of energy and comfort, that we might almost believe the principle of vitality secured to these beings by some especial favour. Such were Parr and Jenkins, and above all, Petrarsch Zortan, who attained the age of 185 years.

Death may take place without palpable disease. Blumenbach believes seventy in one thousand die from what are termed *natural* causes. Harvey found no morbid structure in the body of Thomas Parr.

The transition from senile debility to dissolution—*the last sleep,* as it may be termed—(for the old often die *in* their sleep), is often imperceptible to the closest observation. The system is, in fact, reduced to the lowest ebb, and the lentor of circulation is followed by accumulation of carbon in the blood, which of itself induces sleep.

The phenomena of sleep and dying differ merely in the *duration* of enervation. In sleep, the system is adequate, even while sensation is quiescent, by the integrity of its vital power, to re-accumulate its electricity, there being no disease or extreme asthenia to prevent it. When the system fails in this electrogenesis, death ensues. Sleep and death are thus both the result of exhaustion—the one temporary, the other permanent. Thus we read in the poets of "Death and his brother, Sleep;" "Sleep, thou ape of Death," &c. &c. Sleep, however, is the periodical *remedy* for asthenia, death its *final catastrophe.*

Of sleep old people and children require more than adult age, the former from the natural asthenia of senility and the defect of electricity—the latter from excess of expenditure of this property in the building up of the body, and the absence of mental excitement, so influential in inducing sleeplessness. But this stimulus of exertion must be progressively increased, for we become gradually accustomed to its influence, and, as in the use of alcoholic fluids, require more to exhaust irritability. My friend Dr. ——, like many others of excitable minds, can seldom sleep unless he has expended his electricity by intense exertion. Of course, this must shorten the period of life, and, indeed, he has already endured a most protracted and severe paralysis. This senile sleep of death, unless organic changes exist to interrupt the natural course of decay, may be so painless, even though perfect consciousness be present, that it may seem but a mere *cessation of life,* which may fully justify us in defining death an *intense degree of debility.*

Ere the physiologist commences his research, he must be conscious that there is a property or principle of life, which renders an animal in a great degree uninfluenced by the media in which he breathes, imparted to, or developed in, the germ of a being, and pervading its every fibre. It is the influence of this principle which constitutes vitality—its withdrawal constitutes death.

The *nature* of this principle will probably ever remain a mystery, although very able pathologists have expressed their conviction of the rationality and truth, at least, of the hypothesis imputed to John Hunter, that it consists in electricity; while others, equally learned, have written as much to prove it an idle speculation. Its phenomena do certainly resemble those of voltaic electricity more closely than any other principle; we may therefore fairly presume to adopt the term in reference to the principle of life, especially as that which Hunter and Abernethy conjectured, Breschet and others have seemed to prove, by showing that voltaic influence may not only excite the motive innervation of a muscle, but even elicit secretions from the blood. Its influence in cases of suspended animation, also, is most potent, inducing a far more natural respiration than that produced by mere inflation—the one *raising* the ribs by innervation of the intercostal and other muscles—the other mechanically *puffing up* the thorax by distension.

But whatever the principle of life may be, it is certain (as was proved by Wilson Philip twenty years ago) that one of its sensible agents, innervation, may exist and travel apart from vital tissue; that the function of a nerve may be carried from one cut end of it to the other, still preserving its integrity and force. It is probable, from those experiments, that some subtle and independent fluid must be carried along the inorganic communication placed between the divided extremities of the nerve, and we know that electric matter can be so conducted. It is not, therefore, illogical to believe in a close analogy, at least, between it and the fluid or aura of a nerve. If, moreover, it be so mobile and transferrible, a potent physiological argument is offered in favour of an existence apart from organization.

A very slight blow on a nervous centre will instantaneously annihilate life—a drop of effused fluid will kill the brain, and through it the whole body. If the vital principle were a mere property of structure, would it be so easily dismissed, because an atom of brain is compressed, or when even a metaphysical influence, an *immaterial* cause, has produced it, not the minutest fibre being *perceptibly* changed.

Then, when the heart's muscle is paralysed and ceases to beat, we say the function of the incident nerve is destroyed. But an *artificial* stimulus will for a time reproduce the organic phenomena, as we saw evinced in the heart of Bellingham the assassin, and see constantly in comparative anatomy. Electricity passes into the structure of the body. These stimuli withdrawn, the organ becomes permanently passive—the *muscular irritability* of Hunter, the *vis insita* of Haller, is gone, and innervation ceases; decomposition then speedily ensues, (with all those chemical changes so scientifically displayed by Orfila,) and the larvæ of the *musca tachina*, and other worms, commence their appointed work.

Death, then, appears to be the complete withdrawal of the antiseptic power of vitality, which (from the cadaverous odour commencing some time before dissolution) was in a state of gradual decline, either from defect of innervation, or from poisoned or unhealthy blood—the morbid condition of those two systems, termed by Copland the "chief factors of life."

When the head is cut off respiration ceases—the sensorial influence, essential for natural breathing, does not reach the lung; yet actions are still going on which keep up systemic life. In some cases the pulse in the extremities first ceases—the patient, in the nurse's phrase, *dies upward;* but usually the capillaries, from which the secretions are formed, act long after the lungs have ceased to play, sucking in the already oxygenized blood from the ample reservoirs of the arterial trunks —perhaps even from the lungs; for after an animal has breathed, the air-cells still retain some portion of blood. In trance, probably, where the breathing is scarcely perceptible, the chief supply is from that already passed through the lung.

In senile or natural death, then, life's taper burns out, as it were, at its appointed time, according to the hereditary conditions of the system imparted at the moment of conception or during intra-uterine existence. It is true that nature usually compensates for decline of power, both by deadening sensation and by absorption of superfluous structure, so as not to expend power uselessly, yet debility is ever progressive. The expansive force of growth and deposit ceases; the arteries, therefore, dwindle, ossify, often become completely obliterated; the body wastes, absorption exceeds deposit, and then an exact reverse of the order of growth occurs. The mental phenomena are the first to fail,—the faculty of memory first of all—as was illustrated especially in Sir Walter Scott's decline,—then follow defect and loss of sight, hearing, &c.,—the heart at length, which was the first, being the last organ to act.

We have asserted that this intense debility of age, or death, depends on defect of electricity, inasmuch as its energy may even be restored, not only by those therapeutic agents which impart power, as tonics and stimulants, but by el[illegible]icity, not only inorganic, but, if I may use the term, animal. Thus youth may be said to be *positively*, age *negatively*, electrified. So, when children sleep with old people, or young girls are wedded to old men, wasting and disorder are constantly induced, and there will be by this transference a corresponding energy imparted to the aged. The sacred history of David and Abishag may illustrate this interesting fact.

The closest analogy to senile dissolution is that from extreme syncope, especially in emaciated or exhausted systems. Deficient innervation of the cardiac nerves, which will b[illegible] combined probably with asthenia or anæmia of the coronary arteries, may induce as decided a collapse as paralysis, especially in a heart with a flabby muscle. We have known cases of sudden death somewhat similar to those recorded by Travers, in which presentiment seemed to have produced asthenia of the heart's muscle, and in which that organ was discovered on dissection to be flabby and *uncontracted*, both its auricles and ventricles being totally destitute of blood. Bonetus and Morgagni have related also cases of sudden death from this atony of the heart. The "Medico-Chirurgical Transactions" contain three cases, by the late Mr. Chevalier, of intense and fatal syncope from bloodless heart, to which he gives the name of "asphyxia idiopathica."

But we require not always *material* causes to produce death. Intense mental impression, whether of a pleasing or painful nature, may instantly dissipate the principle of life; for as the sensation from white heat and intense cold is the same, so the physical effect of extremes of emotion are similar. The widow who fell dead at the news of her son's return, was killed by the same proximate cause as those who have died from sudden intelligence of a totally opposite nature. It may seem a remote analogy to assimilate this effect with that of lightning, but in reality both are the discharge of electricity. So also the more gradual influence of the brooding over a prophecy or dream. Travers relates two cases of fatality from these impressions, which induced paralysis of the nervous centres, a total dissipation of the electricity of the body, which is ever after passive and dead.

It would be deeply interesting to inquire more scientifically how these various causes act in dislodging the vital principle, in rendering the body, as the theologian would say, unfit for the residence of the soul. This would indeed be to develop the proximate cause of sleep and death. To do this we must find a nervous centre, from which all vital sympathies emanate, or into which they all converge, the paralysis or death of which would be immediate death to the whole body, as we see exemplified by a full dose of prussic acid. This centre must be believed to be the cerebro-spinal axis. If this be slightly influenced, we should have functional disorder somewhere set up. Thus will the heart's action be often raised ten, twenty, thirty beats in a minute, or the gastric or intestinal functions will be instantly deranged. A merchant in the city, who occasionally consulted me, was instantly affected with extreme vomiting on the receipt of an unfavourable communication. The unchecked or permanently progressive course of this influence, from deranged function to death, would run through the whole range of our nosology.

In our reflections on this cerebro-spinal centre, we must still associate the condition of the blood, especially its excesses either of oxygen or carbon. On it depend those changes of intellectual as well as vital phenomena so characteristic of the dying. In one case we have those exaltations of the mind that seem almost supernatural, while from the influence of the other we have the contrasted symptoms of oppression, as the muttering delirium of typhus, &c., the former being the *irritable* debility, the latter the *torpid* debility of German pathology.

From what we have written, we may presume to conclude that death may ensue from,—

The gradual excitement of living.

The more or less sudden excitement of over-stimulation, shock, disease, or destructive agents.

The absence of stimulation, enervation, hæmorrhage, extreme defect of assimilation, starvation.

Direct sedative, poison.

The condition of dying is marked by very decided signs. Life will often, however, seem at the very lowest ebb, and yet reanimation naturally occur, even after the one slow deep respiration or gasp, so characteristic of the moribund state. I have seen this also combined with such solemn conviction in the mind of the sufferer, as to leave no doubt of approaching dissolution.

A lady (whom I had long watched through the course of deep pelvic abscess) endued with a mind fraught with energy and devotion, was surrounded by her friends, whom she had summoned to her bedside to receive her parting blessing and farewell. On a sudden, when we deemed her at the very verge of death, a thought seemed to pass through her mind, from the intense flashing of her, even then, bright eyes, and she called to me, with unnatural energy, to explain to her the nature of death, gazing on my face as if anxiously listening for my answer. I, of course, replied, that it was one of those mysteries for ever sealed from the intellect of man. Although she was disappointed with this, the stimulus of curiosity, if it were no higher motive, sufficed to call back her declining powers. The skin was still cold, the respiration infrequent and gasping, yet those peculiar diagnostics of dying, the flaccid cornea and the cadaverous odour, were not there. The cornea, indeed, glistened more and more, and seemed to regain its wonted transparency. I from that moment expressed my belief that she would not yet die. She lived ten days longer, so far rallying as to induce me to summon my friend Dr. B. to another consultation.

The infant will often present these phenomena,—it may seem dead, may indeed be laid out stiff and cold, yet, the electricity rapidly accumulating, the lung expands, and the child lives. Even the old crone of a nurse will say of a child, while there is life there is hope ; and indeed we may confidently concur, if we see the cornea still firm and the sphincters unrelaxed.

From the unscientific view of these conditions result those awful instances of premature sepulture recorded in our psychological works. In Bruhier's book we have divers stories of recovery from catalepsy, even during the reading of the funeral service. From our copious budget of these psychological wonders, we may refer to the case of Francois de Civille, who was thrown, at the siege of Rouen, into a state of insensibility, in which state he was carried home by his servant. During a week he became gradually warm, but exhibited no other signs of life. At this period he was flung out of a window by the besiegers, and cast upon a dunghill, where he lay naked for three or four days. Yet even after this, and unaided by the wondrous phial of Renatus, he was restored to life.

A young French lady was condemned, like Juliet, by her father to a hated marriage, while her heart was devoted to another; she fell into a trance, and was buried. Under some strange yet happy influence her lover opened her grave, and she was restored to life and married. Another French girl was even the *subject* of an anatomist, who, on some faint sign of vitality, restored her to life, and made her his wife. All, however, are not so fortunate. Vesalius had opened the thorax of a Spanish gentleman, when the heart forcibly palpitated, but the man of course died. Such was the fate, too, of the Abbé Prevost, and the Emperor Zeno, whose tomb was opened a short time after his funeral, when he was found to have gnawed a portion of flesh from his arm.

The approach of death has very naturally been deemed, even by very wise and pious men, a scene of awful contemplation, as well as a state of agony to the dying. They, however, who have calmly viewed and reflected on this solemn subject, must have concluded, and that without having read Montaigne or Buffon, the first writers on this interesting point, that the Deity has by many merciful modes blunted the dreaded sting of death. We have shown that the natural death of age is but a deep sleep, without consciousness or struggle; and if there be no dread of that "something after death," we may often witness euthanasia, the "babbling of green fields, and looking on the fingers' ends," being the only signs of approaching dissolution. A fit of spasmodic asthma, indeed, will be far more painful.

Sir Charles Blagden died in his chair while taking coffee with Gay-Lussac and Berthollet, and that so calmly, that there was not a drop spilled from the cup in his hand. Dr. Black also died so composedly, that the milk that he was drinking from a spoon was all preserved. Here was evidently an absence of those convulsive efforts, during which Proserpine, as Virgil informs us, cropped the hair of the dying.

In more conscious conditions, true philosophy, combined with piety, can look on death not only with placidity, but even with happiness. I believe Dr. Wollaston watched with scientific interest the gradual failure of his own vital power. Dr. Cullen whispered in his last moments, "I wish I had the power of writing or speaking, for then I would describe to you how pleasant a thing it is to die." Addison summoned the young Earl of Warwick to his bed, to show him how calmly a Christian could die.

Even the death which is caused by violent means, as they are termed, by noxious vapours or gases, drowning, hanging, decapitation, may be not only painless, but pleasurable. We have scientific evidence that the progress towards asphyxia, from the fumes of charcoal, is really an agreeable sensation; and so the curious condition produced by inhalation of nitrous oxyde, as we are assured by Sir Humphrey Davy, from his personal experience. Yet either of these in excess soon deprives an animal of life. Of *sudden* death from carbonic acid, we may believe the same, judging from the placidity of feature. To its *gradual* influence, from which a slow and protracted death ensues, as in the fatal black-hole at Calcutta, we do not allude.

The feelings which precede asphyxia from submersion are still more agreeable. I do not allude to that form, so closely allied to shock or fainting, that has been termed by Desgranges "asphyxia syncopalis;" here, of course, consciousness instantly ceases. I write of the "congestive asphyxia," in which we have all the progressive phenomena of obstructed circulation, in which there are degrees of spasmodic respiration, and poisoned blood is circulating, even when the fingers are excoriated by grasping effort, and the stomach contains muddy water. In these cases, were it not for positive and personal evidence, we should of course believe the converse.

Dr. Adam Clarke illustrates this in his interesting dialogue with Lettsom on the subject of his own submersion, during which he avers, that, "though perfectly conscious, he felt no pain, and even believed himself in a state of bliss in paradise."

While the Humane Society were framing their scientific rules for resuscitation, I remember one pale melancholy girl, who glided in before us like a spectre. She assured us that the sensation of drowning was but an intense feeling of faintness preceding insensibility, but that the *coming to life* was agony—a sensation, it seemed, as of pins and needles in the spinal marrow.

The expression of Clarence, therefore, "My God! methought what pain it was to drown," was but the coinage of Shakspeare's prolific brain. We cannot, however, wonder at the request of those resuscitated, that no attempt be made for that effect, should they again suffer submersion. We can have no doubt, especially with the aid of their analogy, that the pain of being born exceeds that of dying.

Suspension, also, (of course I do not allude to the *instantaneous* death from luxation of the dentatus,) unless it be imperfectly effected, is proved to be painless. The melancholy Cowper has recorded, that in one of his three attempts at suicide, he hung himself over the door of his own room in the Temple, and that his suspension was perfectly painless.

I will quote, also, this passage from Lord Bacon's "Historia Vitæ et Mortis."—"Memini me accepisse de generoso quodam qui ludibundus a curiositate desiderabat scire qualia paterentur in patibulo suspensi, seseque suspendit super scabellum se allevans, et deinde se demittens," &c. "Ille interrogatus quid passus esset? Retulit se *dolorem non sensisse;* sed primo observatum sibi fuisse circa oculos speciem ignis," &c.

We may now be quite prepared to believe that decapitation, as well as a quick division of the spinal marrow by a sword or bullet, is too instantaneous for the recognition of sensation: of this Cabanis and Guillotin have declared their firm conviction.

We have very curious speculations regarding the consciousness of the decollated head. Charlotte Corday's cheeks are affirmed to have blushed at the exposure of her bosom, and the lips of Mary Stuart to have prayed visibly.

The latter fact we may believe on the principle of excito-motory action, as we may the unconscious closing of the eyelids when the head has been held to the sun. Regarding the blushing cheek, we may decide from the experiments of the Heidelburg professor on the head of Sebastian Zink, at Rastadt. When bitters were placed on his tongue, and "pardon" hallooed in his ear at the moment of decapitation, the head was insensible to both.

It may very rationally be asked if groaning and writhing of the body be not proof of suffering? No. In partial apoplexy groans are unconsciously uttered; and those who have groaned deeply when submitted to operation during the influence of æthereal vapour, have declared their utter unconsciousness of pain,—the sensation was often agreeable. Even the convulsive throes of the criminal on the gallows may be involuntary, and uncombined with consciousness. Like the twitchings of adynamic fever, they are the result of mere muscular irritability, and can be imitated by galvanism on the dead body. Thus, too, may a paralysed and unconscious limb be excited into action, the more readily, indeed, as Marshall Hall has beautifully shown, in consequence of the apathetic state of the sensory nerve.

But there are moral or metaphysical causes which tend to mitigate, or qualify, at least, the pains of dissolution, even from the infliction of torture. Different brains have different degrees of sensibility.

The concentration of mental energy, especially if induced by the two great springs of voluntary endurance, religion and honour, is often the source of actions and conduct almost superhuman. Mandrin smiled while his bones were broken on the rack.

Such, too, is the heroism of the American Indians along the banks of the Orinoko, (if Gamilla be correct,) during those probationary tortures for the sovereignty, in which the victim often sinks in mortal agony. The Hindu widow, with the hope of rejoining her husband in the realms of bliss, clasps the corpse in her arms, and placidly burns to death on the funeral pile. To inherit, also, the realms of Brahma, the Fakir clenches his fist for years, until the nails grow through his hand; or forces the hook between his ribs, and whirls himself aloft until he expires; or smiles as he is crushed beneath the wheels of Juggernaut.

The excitement of devotion, also, confers this apathetic or enduring fortitude, illustrated by the death of Cranmer, Latimer, Ridley, and the host of martyrs burned in the reign of Mary Stuart.

Apathetic death may be induced, even in the most sensitive, by the exhaustion of this sensibility—mentally or corporeally. At the request of her judges, it was my duty to watch the young and beautiful Anne G., condemned for infanticide. The convulsive agonies succeeding her condemnation at length subsided into a perfect trance, and with a languid smile she met her fate, as if life and its consciousness had long been parted. Those who have suffered from intense sea-sickness have affirmed their recklessness even of extreme danger during the malady, and the absence even of the slightest alarm, even when their vessel foundered on a rock.

In writing on the Treatment of the dying, we do not intend to allude to those cases of acute disease, or asphyxia, or poisoning, recoverable by remedy. It may be said of many thus affected, that although dying, they do not die: of catalepsy and syncope,—that although in the real semblance of death, yet shall they live. Our precepts will be directed to the sacred duty of strewing poppies on the pillow of the really dying —of calming the progress to the grave.

At that moment which has been termed the *crisis*, the fiat of the Creator may be said to have gone forth. If favourable, it is the triumph of the vital principle over morbid influence, the first step towards convalescence, the next being health. If unfavourable, debility—the first degree of that condition the last of which is death—continues (it may be, with fluctuations) its downward progress. Vital energy more and more flags; the septic change may be said to have already commenced. The sensorium speedily exhibits signs of decay; and finally, its natural consequence, the arrest of the vital functions, closes the scene of existence.

The heathen stoics not only deemed what we call killing and slaying "justifiable homicide," but vaunted suicide as a virtue, believing life to be the *property* of him on whom the gods had conferred it. So Cato and Brutus and Cassius and Anthony fell on their swords, Pomponius Atticus killed himself by starvation, and Hannibal by poison.

But the Christian moralist will of course decide that it is the duty of the physician to prolong the life that God has given, under any circumstances. In cases of monstrosity, it might almost seem justifiable to destroy. But the moral and criminal laws both term the act murder.

In the intense and agonizing state of rabies—hitherto, alas ! remediless—in which the expressive look, perchance the lips, of the patient beseech us to terminate his mortal agony, we should indeed be placed in a painful dilemma, were we not to remember the sacredness of human life, and that remedy, it may be *specific*, may yet be discovered for this distressing malady. To soothe, but not destroy, is our precept; and heartless, indeed, would it be, were we to withhold our ministration when the scene of life is closing on a powerless and suffering creature.

Now the dying state, like that of infancy and protracted senility, is susceptible of very slight impressions. The waste of electricity has reduced the one to the condition of the others—that of direct debility; under which the slightest influence which excites unduly either of the vital systems, may be followed by instant dissolution. Even a look—a word—the motion of a head or hand—may be the source of serious evil. Calmness and quietude should, therefore, be the golden precepts of the sick chamber. All ceremonious display on the part of the physician is not only injudicious, but cruel. Even the mode of calculating the pulse is a point of much importance. The expressions of the physician are often watched with the most scrutinizing attention, and a turn of the eye, or a motion of the lip, may often produce much unfavourable excitement. We may often prove this by the increased frequency of the pulse while our finger is on the wrist. The watch is paraded far too frequently; indeed, except at the period of crisis, or in very peculiar maladies, it may well be unemployed. He must possess a very slight degree of *tactus eruditus* who cannot count the pulse correctly, within five seconds,—and this is sufficient exactness to determine our course in the majority of maladies. At the bedside of the dying, the watch may be discarded altogether.

The expression of the physician's countenance should be cheerful; he should greet his patient with smiles, or in more serious maladies, at least with placidity. Even the important questions regarding symptoms and feelings should not seem serious to the sufferer. Above all, the physician (even for the mere satisfaction of friends,) should ever assume a perfect confidence in his own judgment. Confidence, the conviction that the resources of professional science and wisdom are at hand, impart a charm even in moments of danger, and especially on the sensitive mind and weakened body of the dying.

Regarding the imparting of prognosis to patient or relatives, we must often feel extreme difficulty; yet even this important and solemn duty may be fulfilled with a degree of delicacy and caution which may diminish much of its painful excitement. It may often be judicious, however, to make some firm and cautious relative our confidant, whose mind may be prepared for mortal consequences. This will tend to insure confidence, and, in the end, to console those who have suffered bereavement.

To impart the awful truth that hope of life is fled, requires, of course, the most serious and delicate judgment. We must feel that we are announcing the awful fiat of the Deity to a living soul about to be ushered into his presence. If a silent prayer for Divine aid be ever consolatory or efficacious, it must be while we are thus ministering in the moments preceding so solemn a transit.

The terms and accents in which we express our opinions or our precepts should be carefully modified. The same sentiments may be expressed correctly and intelligibly by several different sets of words; so a patient may be induced to adopt that which is in itself offensive or painful, according to the mode in which it is recommended. To the sensitive mind of the dying, the adoption of the *suaviter in modo* is of high importance: one word may make all the difference—it may be a bane or a blessing. Thus even death may be alluded to as *sleep*,—a draught may be termed an *anodyne* or *cordial*, according to its nature.

The word slumber is in itself an anodyne, and like the lullaby of the nurse, may induce balmy repose even in the dying: so with the gentle terms of endearment, the dying mind may not only be gratified, but delighted. Sympathy, indeed, may be over-acted or officiously intruded. We should have a care; the dying are often very acute, and can detect the false flattery of sycophants from truth and sincerity.

This precept should ever be impressed on nurses and domestics—to avoid all useless and officious ministrations, all idle and frivolous questions, so often adopted as a pretence to care and watchfulness. Even the sobs of unavailing sorrow should, as much as possible, be suppressed or concealed from the dying. Those who have not witnessed and remembered such effect can scarce believe the aggravation of a dying moment from such an influence. A lady whom I was anxiously watching was dying composedly—I may write happily—until she was aroused from her morient slumber by the sobs of her husband. In tones more powerful than we could have believed, and than she had for many days uttered, she exclaimed, (pointing with her skeleton finger to her husband,) "I don't wish to live—only for *him*—only for *him!*" The effort was intense, and was directly followed by most painful rigor, which lasted two hours, and was then followed by extreme flushing and heat, until she at length lapsed into her former state of quiescence. I cannot paint the agonizing regret of the husband at thus being the innocent cause of so much excited suffering in one whom he ardently loved.

In the treatment of the dying, when hope is gone, it would be a cruel process were we to aim at the raising of a pulse, and so, perhaps, increase or protract suffering; yet the even free administration of stimuli, wine, brandy, or ammonia, is often adopted. The delirium, which, from the subsidence of action, had ceased, inducing those calm and lucid moments so often preceding dissolution, is often thus reproduced, and the dying rendered unfit both for the expression of their last wishes, and for that which is at least as important, the last offices of religion. Thus, too, is the quiet sleep of death converted into a spasmodic convulsion; indeed, such is the impression on the mind of the dying, especially in the last stage of phthisis, who have been known to express regret and sorrow at being thus painfully revived from the slumber of death.

It is essential to regard attentively the *breathing* of the dying, the

*effort* of respiration being often the most painful feeling. This is evinced by the sudden throwing back of the arms, or by restlessness, sobbing, or gasping. When these signs are perceived, the position of the patient should be quietly changed, the body raised on the pillow, or the temperature of the chamber reduced, to meet the cause of the dyspnœa. The temperature of the chamber, indeed, should be ever regulated according to the season and the wishes of the patient, if they can be expressed. It will often be eligible to close the curtains, that the dying may not be excited by surrounding light or motion, or the noise and voices of the attendants. Indeed, I would, in many instances, recommend that only one quiet person should remain in immediate attendance in the room, who should sit, as it were, behind the pillow. If the season be sultry, it will be essential to open windows, and strew a light solution of the chlorides about the room, and apply diluted eau de Cologne, or aromatic vinegar, or æther, or other agreeable refrigerants, on the brow and cheeks.

It must be with sensations of horror that we reflect on the savage cruelty which disgraced the last century, and I fear in some districts even in our own time—the dragging of a dying creature from a bed of down to a hard mattress, under the barbaric notion that life would not *pass* unless this were done. It is true, the victim is not now strewed with ashes as in former days, but this inhuman transfer *is* made, and the pillow and bolster often forcibly withdrawn, by which death is, perhaps, intentionally accelerated. The Brahmin who directs the dying Hindu to be laid quietly by the waters of the Ganges was an angel of mercy compared to these executioners.

As the moment of death approaches, the mucous secretions fail, the lips, tongue, and fauces becoming dry and parched. They should then be gently and assiduously moistened with warm vegetable jelly, on a soft feather or sponge, which may often be done without the consciousness of the patient.

When the organic innervation is at the lowest ebb, and the power of deglutition is lost, it will be well to remember that there are other modes of imparting medicine and nutrition. Thus, by the absorbing power of the skin, anodynes may be introduced into the system. Extensive ablution, therefore, with diluted eau de Cologne, or vinegar, may be adopted over the surface [illegible]f the body, and in a more concentrated form in the palms of the h[illegible]ds, combined, if that be essential, with anodynes.

It will be sufficient if we merely glance at the treatment of the *corpse:* yet the *ceremony*, as it is termed, of *laying out* (which is often most unceremonious and degrading), is too often imposed on those who are regardless of all feeling and delicacy. The duty should ever be regulated, if possible, by those to whom profit is secondary to feelings of respect for the dead. The period of this laying out should be protracted to five or six hours, and the body still preserved from the influence of cold air, the nostrils and mouth being kept uncovered, so that not even a remote chance of re-animation may be lost.

There may be many other precepts, or points, of no less importance than those we have written, some of which may, perhaps, be deemed unimportant. But the most trifling act of ministration that may impart comfort to the dying, will ever prove consolation to the hearts of attached friends, and is not unworthy the attention of the most scientific physician.

8, *Storey's Gate, St. James's Park,*
*October*, 1847.

---

### THE IDIOTS OF THE BICÊTRE.

